# Discovering spatial interaction patterns of near repeat crime by spatial association rules mining

**DOI:** 10.1038/s41598-020-74248-w

**Published:** 2020-10-14

**Authors:** Zhanjun He, Liufeng Tao, Zhong Xie, Chong Xu

**Affiliations:** 1 School of Geography and Information Engineering, China University of Geosciences, Wuhan, 430074 China; 2 Artifical Intelligence School, Wuchang University of Technology, Wuhan, 430223 China; 3 School of Geography and Remote Sensing, Guangzhou University, Guangzhou, 510006 China

**Keywords:** Behavioural ecology, Environmental impact

## Abstract

Urban crime incidents always exhibit a structure of spatio-temporal dependence. Exploration of the spatio-temporal interactions of crime incidents is critical to understanding the occurrence mechanism and spatial transmission characteristics of crime occurrences, therefore facilitating the determination of policing practices. Although previous researches have repeatedly demonstrated that the crime incidents are spatially clustered, the anisotropic characteristics of spatial interaction has not been fully considered and the detailed spatial transmission of crime incidents has rarely been explored. To better understand the spatio-temporal interaction patterns of crime occurrence, this study proposes a new spatial association mining approach to discover significant spatial transmission routes and related high flow regions. First, all near repeat crime pairs are identified based on spatio-temporal proximity. Then, these links between close pairs are simplified by spatial aggregation on spatial grids. Based on that, measures of the spatio-temporal interactions are defined and a spatial association pattern mining approach is developed to discover significant spatial interaction patterns. Finally, the relationship between significant spatial transmission patterns and road network structure is analyzed. The experimental results demonstrate that our approach is able to effectively discover spatial transmission patterns from massive crime incidents data. Our results are expected to provide effective guidance for crime pattern analysis and even crime prevention.

## Introduction

Urban crime is a major type of public safety events and closely related with residents’ personal and property safety. Understanding the spatial patterns of crime incidents plays an important role in explaining major factors for crime occurrence and generating strategies for crime prevention. Several previous studies have focused on exploring the spatial distribution of crime occurrence and have reached a consensus that crime occurrence is not randomly distributed spatially^1–4^. On the contrary, the distribution of crime occurrence always exhibits a “spatio-temporal dependence structure.” Actually, the “spatio-temporal dependence structure” of crime can be understood in two ways. On one hand, it indicates that there are significant spatial clusters of crime, regardless of the spatial units of analysis^5–7^. The concentration of crime at one place has been explored by many criminologists and can even be termed as the “criminology of place”^8^. For example, Weisburd et al. (2004) found that approximately 50% of crime incidents over a 14-year period occurred at only 4.5% of the street segment. The “spatial clusters” of crime indicates the fact that both the criminal opportunity and crime occurrence are closely related with “space”. The cluster of crime can be explained by the optimal foraging theory which states that criminal optimize foraging strategies to increase the rate of reward whilst minimizing both the amount of time searching and risk of being caught^9^. On the other hand, “spatio-temporal dependence structure” also demonstrates that the risk of crime is affected by its spatial neighborhood. In other words, if a crime incident is identified in a given area, then the surrounding area may experience an increased risk of similar crime occurrences after a period^4, 10^. In this situation, crime incidents exhibit the space–time interaction and thus the spatial and temporal elements should be considered jointly.

To discover spatio-temporal dependence of crime incidents, there are generally two kinds of strategies. First strategy is operating on the crime incidents directly, including hotspot (or clusters) detection and hotspots prediction. The hotspot detection is commonly used in crime pattern analysis, with aiming to pick up spatial areas of concentrated crime. The commonly used methods for detecting crime hotspots include kernel density estimation (KDE), spatio-temporal scan statistics and spatial statistics such as Ripley K and Getis-Ord Gi*^11–14^. These approaches are not limited in Euclidean space, but also can be applied to network distance. However, the spatial hotspot detection method still suffers from some limitations^14,15^. First, theoretical explanations of underlying causes of crime hotspots have not been fully developed, barring some efforts to establish the link between crime concentration and related criminal theories (e.g. such as social disorganization)^16^. Second, hotspot identification is usually based on the historic data, not performing well for crime risk prediction because spatial hotspot of crime incidents will change over time^17,18^. To better understand future crime risk, researchers also try to model the crime patterns mathematically and then predict the crime hotspot and risk. Because crime incidents take the form of events that occur at discrete points in space and time, they are usually modelled by the spatio-temporal point process (*STPP*)^19^. One of the typical model is the self-exciting point process (*SEPP*), which assumes that the crime points can be classified as *background* and *triggered* events^20^. The relationship between background and trigger points can be modelled by in a mathematical way. Then, based on the mathematical model, crime clusters or crime risk can be predicted on specified spatial grids or street network^21,22^. To further improve the prediction performance, Rosser and Cheng even try to model the spatial isotropic into the SEPP model^22^. Due to its prediction performance, the SEPP model even has been developed commercially (as *PredPol*) and applied in some countries.

The second strategy to explore spatio-temporal dependence concerns on the “near repeat” crime^10,14,23^. The near repeat phenomenon suggests that when a crime occurs in a specific location, the area surrounding that location may experience an increased risk of a similar crime occurring for a distinct period of time^4,10^. It should be noted that near repeat phenomena do not ensure a series of crime conducted by a single criminal. However, from the spatial perspective, near repeat crime indicates the “spatial interaction”. Such spatial interaction can be related both “spatial heterogeneity” and “environmental similarity”, which explains why certain places experience more crime events and what boosts the near repeat crime^24,25^. Since the near repeat crime concerns more about the interaction in spatio-temporal proximity, making it possible to predict future victimization^26^. Currently, research on near repeat crime mainly deals with two issues: checking the generalizability of the near repeat phenomenon and determining to what extent it can help to predict the crime. Much of the extant research investigating repeat victimization has focused on the crime of burglary^15,27^. To assess the generalizability of near repeat victimization, researchers have explored the near repeat phenomenon related with other types of crimes, such as shootings, theft from motor vehicles, and even insurgent activities^10,28,29^. On the other hand, quantitatively measuring the spatial and temporal ranges of near repeat crimes is quite instructive for police practice decision. In general, the crime risk will be significantly higher within a short spatial and temporal range of an initial victimization and will exhibit a clear spatio-temporal decay effect beyond the spatio-temporal proximity. A widely used tool for identifying near repeat victimization patterns is the Near Repeat Calculator (NRC)^30^. The NRC can tell whether there is a significant near repeat victimization pattern in specified spatial and temporal range. The principle of the NRC is the Knox test, which calculate the difference of observed near repeat crime pairs in a spatio-temporal range with expected number by chance^27^. Recently, researchers try to examine the extent to which near repeat patterns can prevent crime^31,32^. They found that crime hotspot and near repeat crime are not co-located with each other and significant space–time clustering does not necessarily indicate an actionable near repeat problem. Their findings suggested that a global near repeat pattern is not sufficient to quantify the crime prevention values. The global level of space–time clustering revealed by the NRC just the first step to understanding near repeat patterns^31,32^. In fact, the spatial interaction of near repeat crime would exhibit the “slippery” manner^33^. For example, Johnson and Bowers mapped all “pairs” of near repeat crime on different spatial regions and then calculated correlations for number of “close pairs” in different months. The results proved that the near repeat crime pairs showed a “slippery manner”, instead of keeping stable in space. However, how near repeat phenomena move in space is still a remaining question^34^.

To better reveal the space–time interaction of crime occurrences, the anisotropic characteristics of spatial interaction must be considered. This study aims to discover the significant spatial interaction patterns embedded in the near repeat phenomena (termed as “spatial transmission patterns”). In addition, it also tries to discover the “high flow regions” related with the spatial transmission. These “high flow regions” are defined as “source” or “sink”, which are inspired by concepts in ecology^35,36^. To be more specific, the “source” represents spatial regions from with enough objects flowing out. Conversely, the “sink” represents spatial regions a lots of objects entering it^36^. Both high flow regions and spatial transmission routes can be discovered by approach of spatial association rule mining^37^. Therefore, this paper proposes a framework to discover spatial interactions patterns of near repeat crime by using spatial association rule mining.

The main contributions of this study lie in the following aspects:

1. Some new concepts are defined to study spatial interaction patterns of crime with fully considering the anisotropic characteristics. In this study, we borrow concepts in ecology and spatial data mining to model the dynamic characteristic of near repeat phenomena. The spatial interaction between different regions are modelled as spatial transmission routes and the regions with high flow are modelled by “sources” or “sinks”.
2. A new spatial data mining approach is developed to discover the significant spatial interaction patterns. First, we define some indicators to model the spatial association strength. Then, based on these indicators, algorithm of mining spatial transmission patterns is developed. The proposed approach can be applied to network structure and discover dominant regions and interaction links between regions.

The rest of the paper is organized as follows. In "Materials and methods", the proposed methods, the study area, and study data are described in detail. In "Results and discussion", the experimental results and discussion are presented. Finally, we summarize the advantages and limitations of the current study in the last section.

## Materials and methods

### Framework for discovering significant spatial transmission pattern of crime occurrence

**Figure 1:**
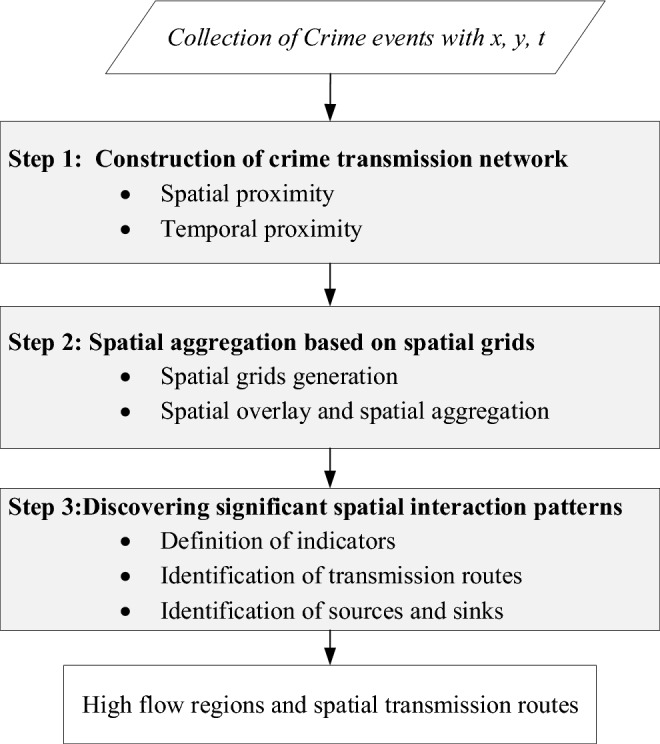
Overview of framework for discovering spatial transmission patterns of crime occurrence.

In this section, a framework for discovering significant spatial interaction pattern of crime is developed. As illustrated in Fig. 1, the proposed framework comprises the following three steps.

The proposed method works on a collection of crime points with spatial and temporal information. Firstly, near repeat crime pairs are identified by specifying the spatio-temporal proximity. All near repeat crime pairs would form a network structure, making it difficult to discover the dominant patterns. Therefore, we simplify the network by overlaying with spatial girds and then aggregating it. Finally, some indicators are defined to measure the spatial interaction strength, and a spatial association pattern mining approach was developed. The whole framework is designed to discover the most probable spatial transmission routes and related high flow regions. Explanation for each step is further illustrated in following sections.

#### Construction of crime transmission network

This study aims to discover spatial interaction patterns from a collection of discrete points. Each point represents a location where crime incident happens. However, these crime incidents are not totally independent, but related with each other in spatial aspect. The typical phenomena demonstrating such interaction is the near repeat crime. The interaction between near repeat crime pairs can be represented as a “directed link”, and a directed network can well describe the spatial interaction of all crime incidents (denoted as “transmission network”).

The crime transmission network is composed of a node set *V* and an edge set *E*, which can be denoted as *N* = (*V*, *E*). Each node in *V* indicates a crime incident and each edge represents the spatio-temporal relation between two incidents. Because the influence of a crime only existed in a limited spatial and temporal range, spatio-temporal proximity should be defined to identify the near repeat crime. Specifically, given two crime incidents c_1_ and c_2_ occurring at timestamps *t*_*A*_ and *t*_*B*_, their spatial distance and time difference are denoted as *r*_*AB*_ and *t*_*AB*_, respectively. A directed edge *e*_*AB*_ is added if the following conditions are satisfied:

1 $$\left\{ {\begin{array}{*{20}l} {{0} \le t_{B} - t_{A} \le \Delta t} \hfill \\ {r_{AB} \le \Delta s} \hfill \\ \end{array} } \right.$$

where Δ*s* and Δ*t* are two parameters to define the spatio-temporal proximity. In this manner, a crime transmission network can be constructed with the dual constraint of spatial and temporal proximity.

#### Spatial aggregation based on spatial grids

**Figure 2:**
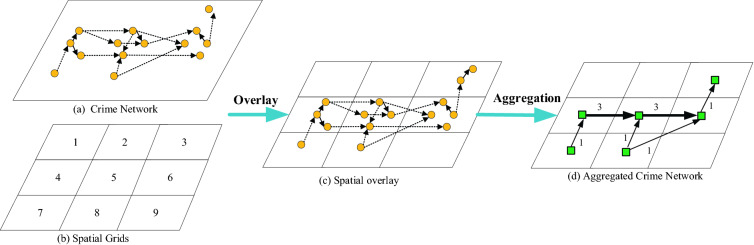
Illustrative example of spatial aggregation of original network.

In the crime transmission network, each edge stands for an instance of near repeat crime pairs. As described above, crime transmission network indicates the “spatial interaction”. To explore the spatial interaction, the spatial analysis scale should be determined first. On the other hand, because “near repeat” pairs are judged by the spatio-temporal proximity, a single crime incident may be viewed as “close pair” with many other incidents, all the “close pairs” of crime incidents may form a complex structure (like a complex network), thus making it difficult to extract dominant patterns from such complex structure. As illustrated in Fig. 2, network nodes are usually clustered and network edges are usually intersected in an unregularly way. In situation of lots of nodes and edges, it is difficult to extract dominant spatial interaction patterns from the complex network.

To address the above issues, we then overlay the crime transmission network with spatial grids. The advantage of applying spatial grids lies in two aspects. First, the spatial interaction should be explored at a spatial scale. The analysis scale is closely related to spatial grid size. By setting different grid sizes, multiple scales analysis results can be achieved. Second, by overlaying spatial grids with the crime transmission network, each node and edge in the network can be associated with one or several spatial grids, then the crime network can be simplified greatly by spatial aggregation. As an example illustrated in Fig. 2, each circle in sub-figure (a) represents a crime incident, and crime pairs are connected by dashed lines. Obviously, it is not easy to identify the dominant spatial patterns. The complex network can be simplified by overlaying with spatial grids. The close crime pairs can be classified into two categories: “following in same grids” and “crossing different grids”, and those crossing different grids can be used to analyze spatial interaction between different regions. In sub-figure (d), each spatial region is represented as a square, and the numbers beside links represent number of close crime pairs crossing different regions (i.e. the by spatial aggregation). In this manner, the original crime transmission network has been simplified. It should be pointed out that the “spatial aggregation” does not discard any close crime pair. Those falling in a single grid can be used to measure strength of spatial interaction, which will be described in following section.

#### Discovery of significant spatial interaction patterns

From the above description, we can learn that the aggregated crime network is a directed network. Each node of network represents a spatial region (spatial grid) and edges indicates near repeat pairs crossing different grids. After the aggregated crime network is obtained, the spatial association rule mining technique can be applied to discover the spatial interactions patterns. The spatio-temporal association rule mining approach is a powerful tool for discovering the interdependence relation in both spatial and temporal domains. The existing research has proved that it can not only reveal a spatial dependence structure among various spatial features or spatial objects^38,39^ but also discover the dynamic interactions among different spatial regions^37,40,41^. For example, Verhein and Chawla describe spatial interaction patterns between different regions using spatio-temporal association rules^37^.

In this study, we also try to summarize the spatial interaction pattern by applying spatio-temporal association rules mining. To fulfil that, following definitions are first clarified.

##### **Definition 1**

Given two adjacent spatial grids (denoted as *G*_*A*_ and *G*_*B*_) and two crime incidents (c_1_ and c_2_), if c_1_ falls in grid *G*_*A*_, c_2_ falls in *G*_*B*_, and their distance satisfies the spatio-temporal proximity constraint in Eq. (1), then the pair of c_1_ and c_2_ is called *an instance of flow* from *G*_*A*_ to *G*_*B*_ and denoted as: *instance* (*G*_*A*_ → *G*_*B*_). The total number of *instance* (*G*_*A*_ → *G*_*B*_) is called the *out flow number of* (*G*_*A*_) and denoted as *outNum*(*G*_*A*_). Correspondingly, total number of *instance* (*G*_*B*_ → *G*_*A*_) is called the *inflow number of* (*G*_*A*_) and denoted as *inNum*(*G*_*A*_). In addition, the total number of close pair which totally falls in grid *G*_*A*_ is denoted as *statbleNum* (*G*_*A*_).

##### **Definition 2**

The spatial region *G*_*A*_ is termed as a *source* when out flow number *outNum* (*G*_*A*_) is higher than random assumption. Conversely, region is termed as *sink* if inflow number *inNum* (*G*_*A*_) is higher than random assumption. A *thoroughfare* is a region which meets both the source and sink requirements. Collectively, *sources*, *sinks* and *thoroughfares* are called *high flow regions* in which near repeat crime pairs can be frequently observed.

##### **Definition 3**

*High flow regions* and *transmission routes* together can describe spatial interaction pattern between different regions. For regions *G*_*A*_ and *G*_*B*_, if the number of *instance* (*G*_*A*_ → *G*_*B*_) is higher than random assumption, then it is called a *significant transmission route* from *G*_*A*_ → *G*_*B*_, denoted as *route* (*G*_*A*_ → *G*_*B*_), while *G*_*A*_ is called *antecedent* and *G*_*B*_ is *consequent* of the route.

##### **Definition 4**

Another two concepts are defined to evaluate the discovered spatial transmission routes. The *spatial support* of a transmission route *r*, denoted as *Sup*(*r*), is the sum of spatial areas referenced in the antecedent and consequent of the transmission route. The *confidence *of a transmission route *r*, denoted as *Conf* (*r*), is defined as the ratio of number of *instance* (*G*_*A*_ → *G*_*B*_) to number of instances flowing out and falling in the antecedent grid. They can be represented formally as:

2 $$Sup\left( r \right) = area\left( {G_{A} } \right) + area\left( {G_{B} } \right)$$

3 $$conf\left( r \right) = \frac{{\sum {instance} \;\left( {G_{A} \to G_{B} } \right)}}{{outNum\left( {G_{A} } \right) + stableNum(G_{A} )}}$$

The first three definitions are used to discover the spatial interaction pattern, while the last one can be used to evaluate the discovered results. The definition of *spatial support* considers spatial semantic of discovered pattern (the size of spatial area) and *confidence* indicates the transmission possibility between *antecedent* and *consequent* regions. Both *support* and *confidence* indicators are commonly used in Apriori-like association rule mining approaches^42^, while these concepts have different meanings in this study.

Based on the above concepts, spatial interaction pattern can be discovered. In spatial association pattern mining process, thresholds for indicators measuring association strength should be determined in advance, e.g. *outNum* and *inNum* in this study. However, determination of the thresholds objectively is not easy. Thus, the discovered results are evaluated via the Monte Carlo (MC) testing. In another words, we aim to find out these patterns with their indicators significantly higher than that would be observed by chance. In the current study, MC methods are employed to generate *N* simulated spatial crime distributions with permutation of temporal information. For example, statistical significance of spatial transmission route *r* can be calculated as:

4 $$p\left( r \right) = \frac{{\sum {\left( {instance\_num^{obs} \left( r \right) \le instance\_num^{ith\_sim} \left( r \right)} \right)} + 1}}{N + 1}$$

where $instance\_num^{obs} \left( r \right)$ represent the number of *instance* (*r*) calculated on real observed data, and $nstance\_num^{ith\_sim} \left( r \right)$ represent the number calculated on a simulated spatial dataset. Then, given a significant level *α* (0.05 by default), if the *p*(*r*) value is less than the significance level, it can be treated as a significant pattern.

### Study area and material description

**Figure 3:**
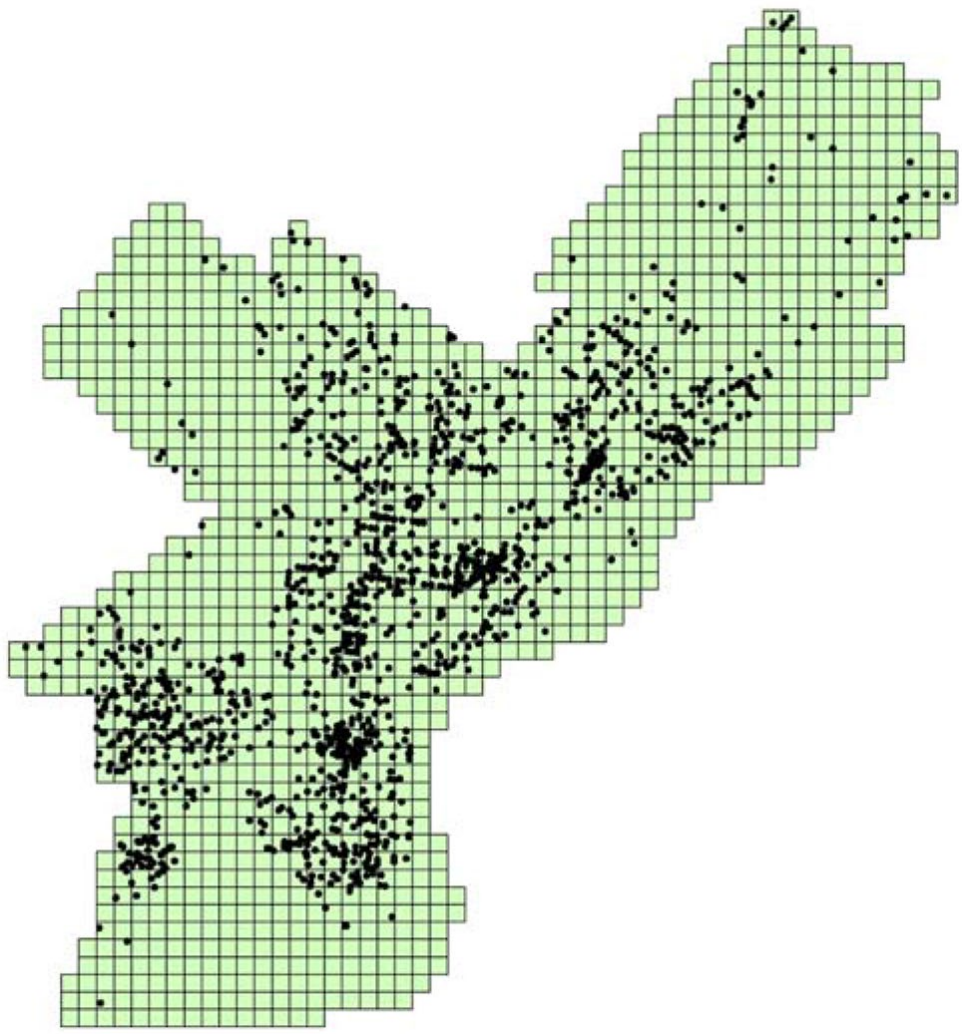
Study region and distribution of robbery incidents.

To evaluate the effectiveness of the proposed approach, we aim to explore the spatial interaction pattern of a robbery in the city of Philadelphia, United States. Located in southeastern Pennsylvania, Philadelphia is an economic and cultural anchor of the greater Delaware Valley, with a population of 1,580,863 (based on 2017 census-estimated results). The crime occurrence in Philadelphia consistently ranks above the national average, which is a major concern for the government. The crime-related data can be freely accessed via the OpenDataPhilly website (https://www.opendataphilly.org/), which provides both crime datasets and basic geographic data. The geographic data include administrative division and road network. The crime incidents are recorded with detailed longitude, latitude and timestamps. In this study, we mainly focus on unarmed robbery during the period of January 1st, 2016, to June 30th, 2016. During this period, the total number of unarmed robberies was 1612. We selected robbery crime as a case study because robbery is frequently observed in the study regions and have a profound effect on the quality of life in urban neighborhood^43^. This study aims to find out: (1) whether robbery crime exhibits the near repeat phenomena? and (2) what kinds of spatial interaction patterns are embedded in the near repeat phenomena? The study region and distribution of robbery crime are showed in the Fig. 3.

## Results and discussion

In this section, near repeat crime pairs are first calculated. Then, spatial interaction patterns are explored based on the proposed method in the "Framework for discovering significant spatial transmission pattern of crime occurrence". Finally, the effectiveness of proposed method is proved by comparing with spatial hotspots and analyzing spatial association with road network.

### Detection of near repeat pattern

**Figure 4:**
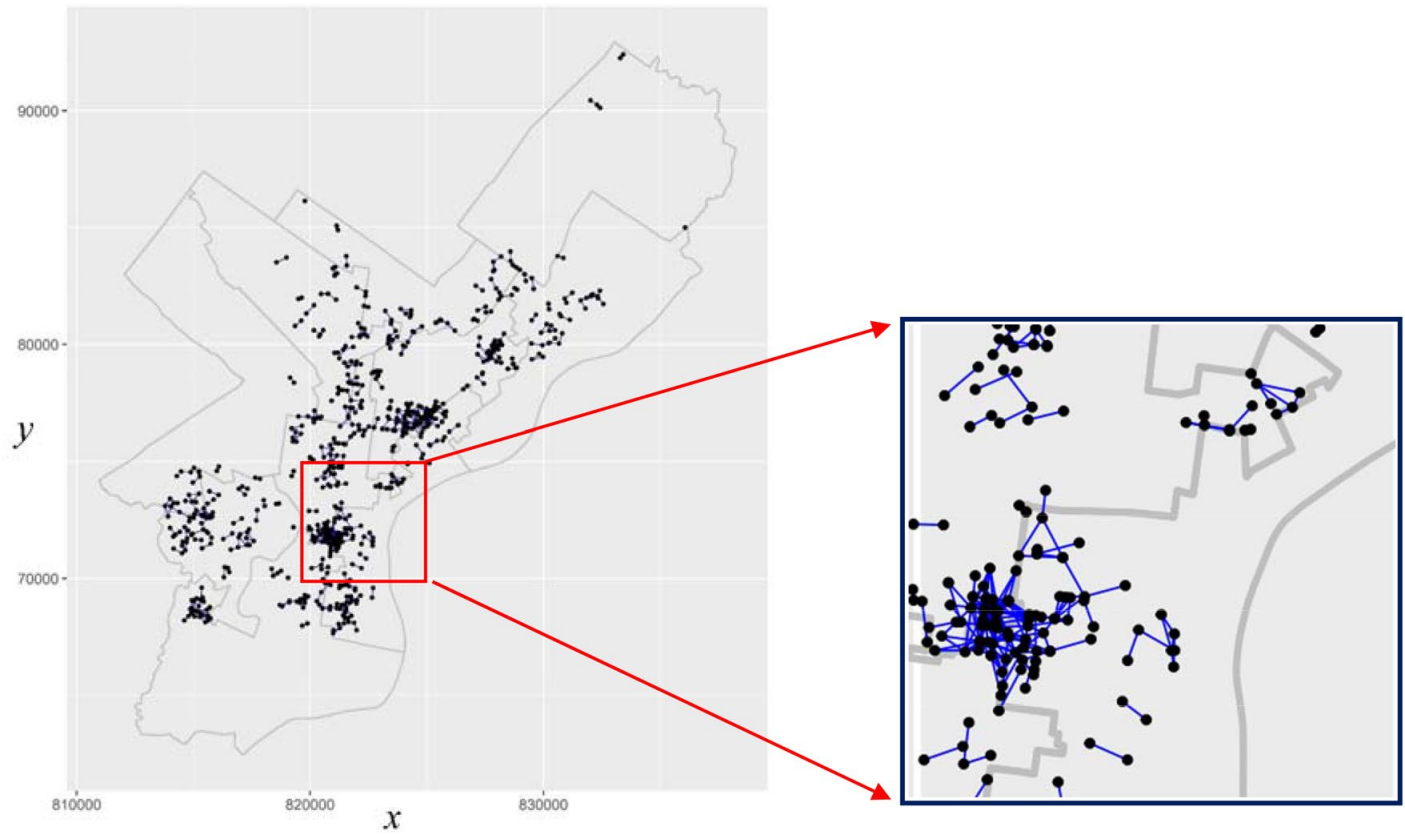
Distribution the near repeat crime pairs.

Firstly, the near repeat patterns of robbery are analyzed. As illustrated in above, the near repeat crime does not ensure a series of crime conducted by a single criminal, it is mainly defined by spatio-temporal proximity. Therefore, spatial and temporal distance need to be specified first. Thresholds for spatio-temporal proximity are related with analysis scale or prior knowledge. In the situation of no prior knowledge, some spatio-temporal statistics can be used to determine the spatio-temporal clustering range, for example, the spatio-temporal K function. Then, determination of spatio-temporal proximity can be determined by referencing the clustering range. In the experiment, the spatio-temporal K function is used to analyze the clustering range^14^. Based on the detected clustering range, the spatial threshold is set as 500 m and temporal threshold is set as 168 h (i.e. a week). Of all the possible combinations, 798 of them are satisfied the spatio-temporal constraint, i.e. there are 798 near repeat pairs in total. For better visualization effect, each near repeat pair is linked by an undirected segment, which is shown in Fig. 4. It is clearly very different to pick out the interesting patterns from Fig. 4. That is due to the spatio-temporal clustering characteristic of crime. Since the near repeat crime is judged by spatio-temporal proximity, the distribution of near repeat crime will also show clustering tendency. In addition, a single crime incident may be paired with several incidents, resulting in the complex structure of the network, as shown in the local region (specified by the red rectangle) in the Fig. 4.

The near repeat pattern can be detected by the Near Repeat Calculator (NRC). The NRC requires the users to specify several parameters, including spatial bandwidth, temporal bandwidth, and numbers of spatial and temporal bands. Then, for each combination of spatial and temporal bands, the observed crime incidents are calculated and the deviation from the random assumption is evaluated by Monte Carlo testing. Following the common practice, the spatial and temporal bandwidths are set to 200 m and 7 days in the experiment, respectively. Both the numbers of spatial and temporal bands are set to 10. The results indicate that a significant near repeat victimization pattern can be identified within approximate 400 m and 7 days. The conclusion drawn by NRC conforms to the finding in our experiments.

### Discovery of spatial interaction patterns

**Figure 5:**
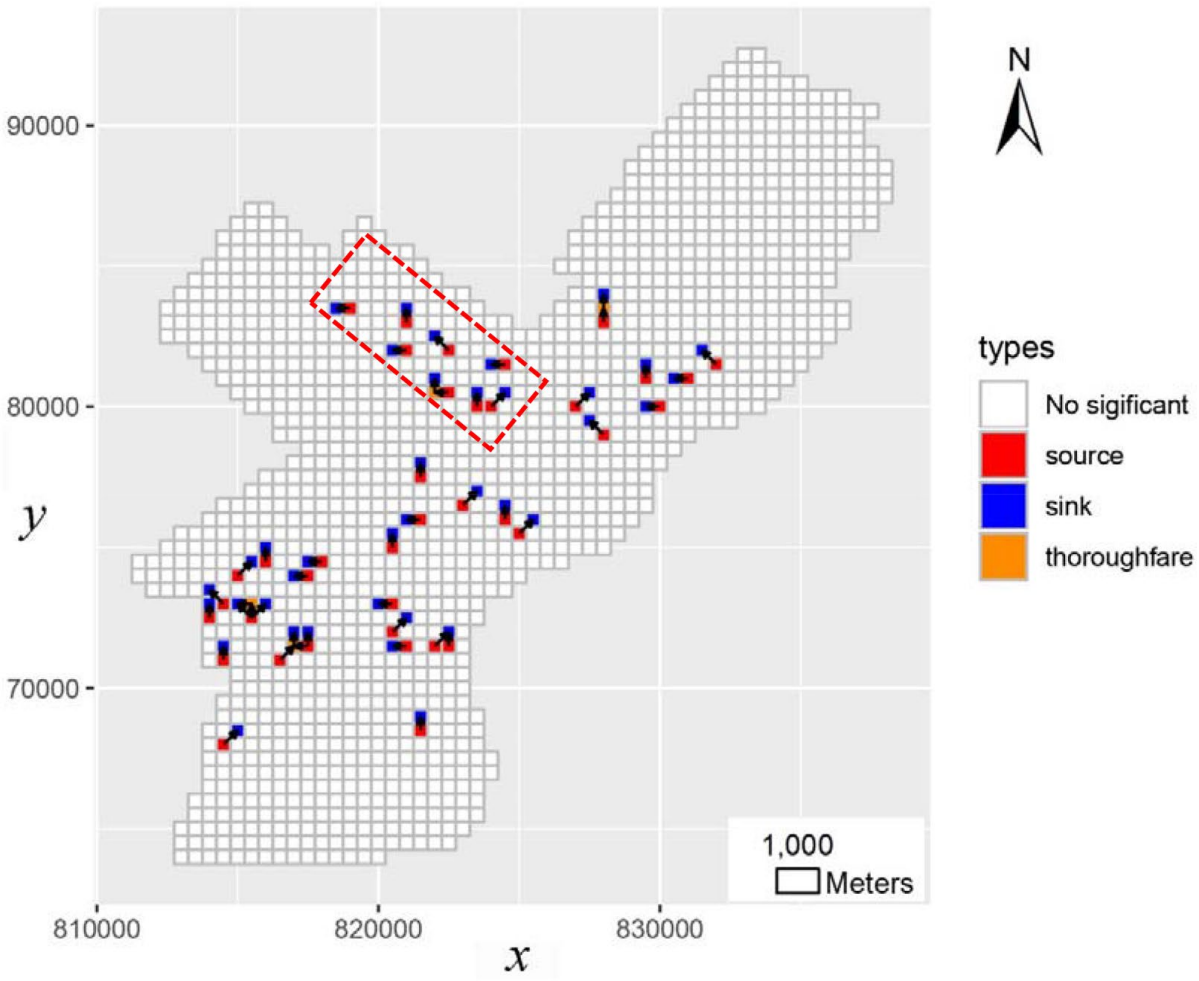
Significant spatial transmission routs and high flow regions.

As illustrated in above, it is hard to pick out the interesting patterns directly from the near repeat crime network. Although NRC can identify the spatial and temporal ranges in which near repeat crime significantly clusters, it cannot explain the spatial interaction pattern of crime occurrence, i.e. how does the crime occurrence transfer in space. To address this issue, the proposed approach is applied to explore the spatial interaction pattern of the robbery crime. First, the near repeat crime network is simplified by spatial aggregation based on spatial grids. Because this study aims to explore spatial interaction patterns based on near repeat crime, the spatial grid size should be related with spatial proximity for identifying near repeat pairs. In addition, the crime occurrence is closely related with physical environment, influence of spatial features in physical environment should as be considered. Empirical research has indicated that features of the physical environment exert the most reliable influence over a street block or two, corresponding to 100–500 m^44,45^. For the above two reasons, the spatial grid size is set as 500 m in the experiment. Then, significant spatial transmission routes and high flow regions are discovered by the approach described in “Discovery of significant spatial interaction patterns” section. During the period, the number of simulation in Monte Carlo testing is set as 99. The final discovered result is shown in Fig. 5.

As illustrated in Fig. 5, the proposed method can discover the high flow regions, which are flagged as “source” and “sink”. These regions indicate the place where near repeat crime opportunity is statistical higher than random assumption. The spatial contexts of these regions may be more suitable for robbery occurrence or criminal flee, and more attention should be paid to these regions in crime prevention. Correspondingly, the transmission routes are marked with arrows from “source” to “sink”. In the experiment, more than 40 routes are identified. The distribution of transmission routes is not as clustered as crime incidents, which may indicate the universality of near repeat phenomena in the study area. Although we also defined the “thoroughfare”, “thoroughfares” are seldom identified in the study area. It manifests the fact that the spatial interaction of robbery only exists in a short distance range, which is consistent with the findings related with near repeat phenomena^46^.

**Figure 6:**
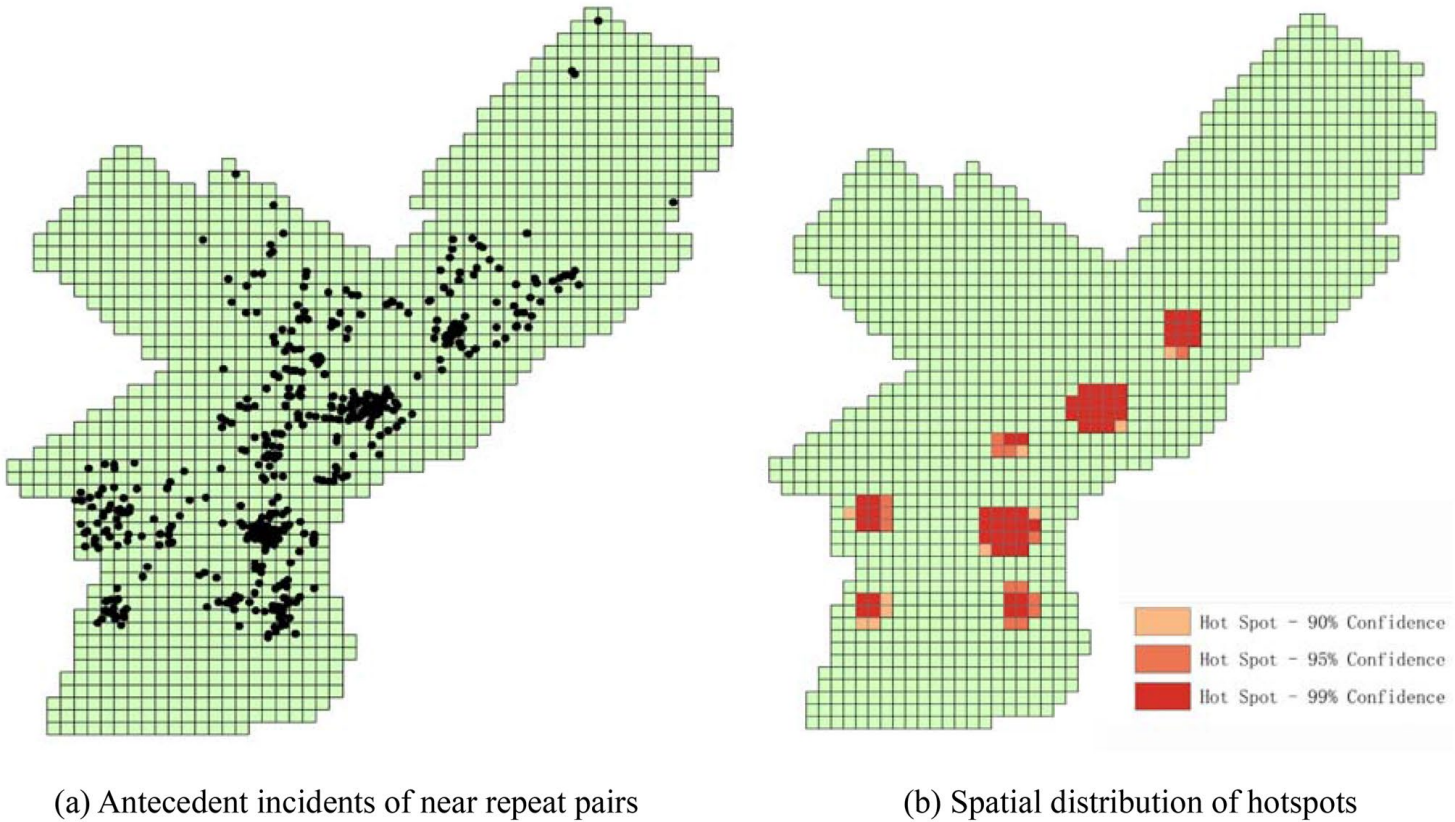
Spatial hotspot distribution of near repeat pairs.

Then, we compare the proposed approach with traditional hotspot detection method. The hotspot detection method (e.g. the Getis-Ord Gi* statistic) aims to find significant “spatial regions” of concentrated crime. The traditional “hotspot detection” approach is based on original crime incidents while the proposed approach concerns more about the “near repeat pairs”, it is unreasonable to compare two methods directly. To fill that gap, all near repeat crime pairs are selected first and then the hotspot detection method is applied to “antecedent incident” of those pairs. In this situation, the spatial hotspot detection method can reflect spatial interaction of near repeat phenomena to some extent. The distribution of antecedent incidents is shown in Fig. 6a, and corresponding hotspot is shown in Fig. 6b. It can be learned that Getis-Ord Gi* statistic tends to identify large and squared regions, because the significance of a feature is determined by both itself and the values surrounding it. Therefore, some small regions may be neglected. By comparing Fig. 5 with Fig. 6b, we can learn that the proposed method can identify both “spatial interaction” and “high flow regions” (i.e. the sources and sinks) in a finer granularity. There are also some locations not reflected by spatial hotspots, for example, the region in the north spotted by a rectangle in Fig. 5.

**Figure 7:**
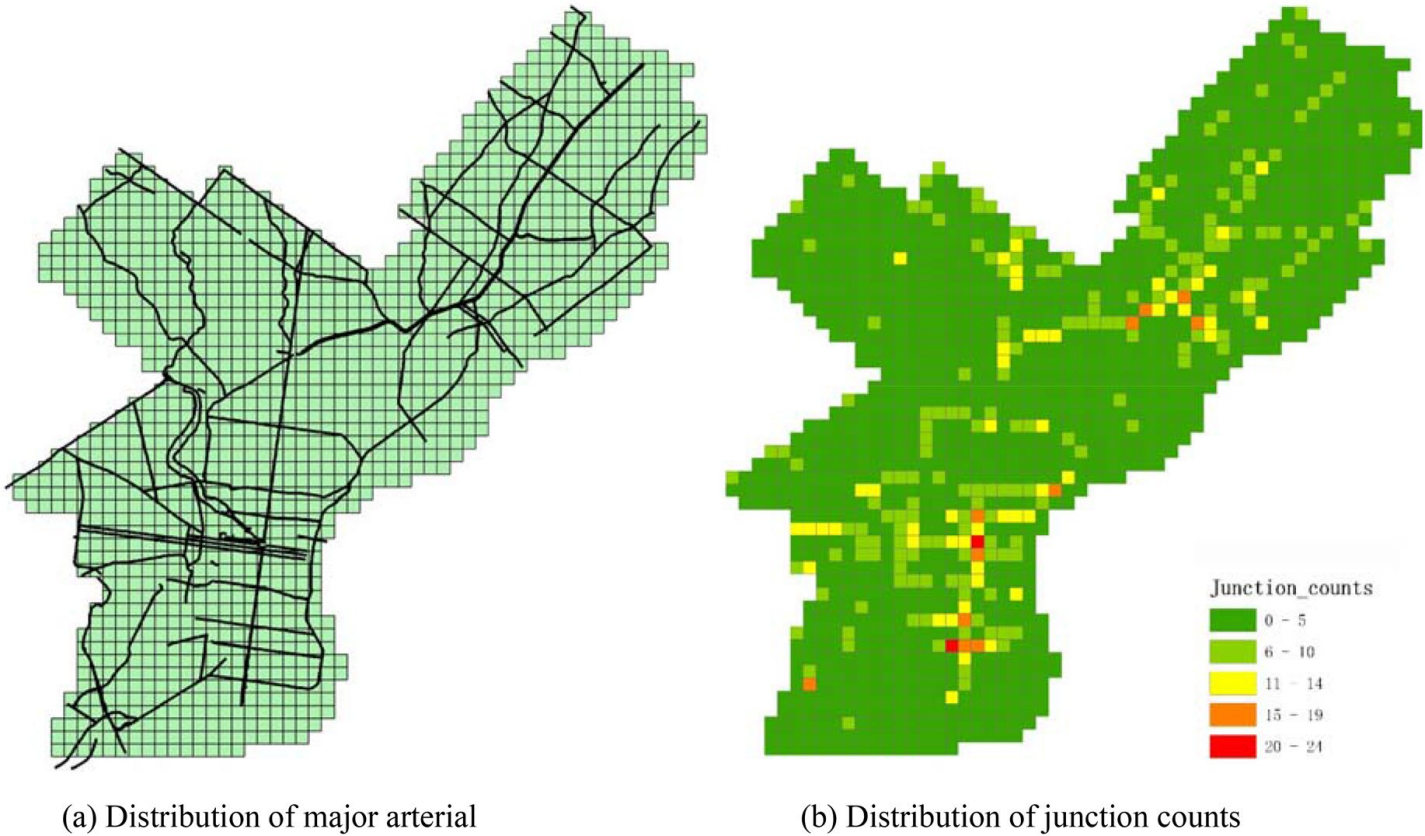
Spatial distribution of major arterial and junction counts.

To prove the validness of findings by proposed approach, we also explore the candidate associated factors for discovered spatial interaction patterns. In the experiment, we select three types of road, including the expressway, major arterial, and minor arterial and then compute the spatial association between road structure and the discovered patterns. To fulfil that, road network is related with spatial grids first and then numbers of road junctions falling each grid are calculated. For example, the spatial distribution of major arterial and junction numbers are shown in Fig. 7. During the spatial interaction discovering process, we have calculated several attributes for each grid, including the outNum, inNum and statbleNum. The correlation coefficient between these attributes and junction numbers can be calculated. The correlation coefficient can indicate the spatial association between road structure and spatial transmission patterns. Of all the select road types, we find that the transmission of robbery crime is closely related to the major arterial. The correlation efficient is about 0.15, not a very high value. However, the correlation is statistically significant by Monte Carlo testing via simulating distribution of road junction numbers. Besides, the association is also can be checked by visual judgment in a qualitative way. By comparing the Figs. 5 and 7, it can be learned most of our discovered spatial transmission routes are located besides the major arterial. For example, the dash line rectangle in Fig. 5 in fact fits well with a major road direction. This finding also can be explained by the optimal foraging theory, which states that animals optimize foraging strategies to increase the rate of reward whilst minimizing both the amount of time searching and risk of being attacked by others^9,33^. Similarly, criminal would commit a robbery and then seek target in neighborhood, if the likelihood of obtaining valuable benefit and adequate escape routes are available. The effect of major arterial on robbery crime may be reflected in two folds. On one hand, it provides great accessibility for non-residents entering the space. On the other hand, it is more convenient for the criminals running away after them committing some robbery activities. This is also consistent with some previous research, which states that a space with high permeability would increases the crime risk^47–49^.

### Discussions

Previous studies have proved the existence of near repeat phenomenon for several crime types, including burglaries and shootings. Most of these studies attempted to identify the spatial and temporal ranges of near repeat phenomenon while assuming the isotropic influence of crime occurrences. By using a case study of a robbery in the city of Philadelphia, this study explores the spatial interaction patterns of crime occurrences by using network analysis and the spatio-temporal association rules mining technique. The results in this study can reveal both the significant spatial transmission routes and detailed local regions where crime incidents transfer with high probability. By associating the discovered results with city infrastructure (e.g. road networks), we can conclude that the major arterial will have an important impact on near repeat crime pattern. The proposed approach can not only serve as an important supplement to existing analysis tools (e.g. the Near repeat Calculator) for near repeat phenomena, but also effectively guide the decision of crime prevention strategies. By identifying high flow regions (i.e. the sources and sinks) and the significant crime transmission routes, the policing resources can be reduced greatly and crime prevention strategies can work better.

## Conclusions

The distribution of crime incidents always exhibits a dependence structure in spatio-temporal proximity. Exploration of the spatio-temporal interaction of the crime incidents, especially the high flow regions and dynamic spatial transmission pattern, is critical to crime control and crime prevention. To better understand the spatio-time interactions of crime, this study developed an approach aiming to identify significant spatial transmission routes and related high flow regions. First, a crime transmission network is constructed with considering the spatio-temporal influence of crime incidents. Second, to simplify the structure of the crime network, the original crime transmission network is spatially aggregated based on the spatial grids, which can be easily achieved on multiple spatial scales. Finally, a new approach is developed to discover significant spatial interaction patterns of crime. The proposed approach can identify significant spatial transmission routes and high flow regions (sources and sinks) related with spatial interaction. Although the experiments mainly focus on a case study of robbery in Philadelphia, the proposed approach can be easily extended to examine other types of crimes in different regions. The discovered spatial transmission patterns can be closely associated with the city’s infrastructure, and it can be explained by the theories of criminal geography (e.g. the optimal foraging theory). The proposed approach can not only discover spatial transmission patterns from massive crime incident data, but also effectively guide crime pattern analysis and crime prevention. In future, the complex association between crime patterns and multiple facilities (e.g. schools, hospitals) should be explored to find out the “spatial scene” or “spatial configuration” for crime occurrence.
